# Severe Enterovirus Infections in Hospitalized Children in the South of England: Clinical Phenotypes and Causative Genotypes

**DOI:** 10.1097/INF.0000000000001093

**Published:** 2016-06-28

**Authors:** Hans de Graaf, Emanuela Pelosi, Andrea Cooper, John Pappachan, Kim Sykes, Iain MacIntosh, Diane Gbesemete, Tristan W. Clark, Sanjay V. Patel, Saul N. Faust, Marc Tebruegge

**Affiliations:** From the * Academic Unit of Clinical & Experimental Sciences, Faculty of Medicine, University of Southampton, Southampton, United Kingdom; † Southampton NIHR Wellcome Trust Clinical Research Facility, ‡ Department of Infection, University Hospital Southampton NHS Foundation Trust, Southampton, United Kingdom; § Public Health England Microbiology Services, Southampton, United Kingdom; ¶ Department of Paediatric Intensive Care, University Hospital Southampton NHS Foundation Trust, Southampton, United Kingdom; ‖ Institute for Life Sciences, University of Southampton, Southampton, United Kingdom; ** Department of Paediatric Immunology & Infectious Diseases, †† NIHR Southampton Respiratory Biomedical Research Unit, University Hospital Southampton NHS Foundation Trust, Southampton, United Kingdom; ‡‡ Global Health Research Institute, University of Southampton, Southampton, United Kingdom; and §§ Department of Paediatrics, The University of Melbourne, Parkville, Australia.

**Keywords:** enterovirus, myocarditis, sepsis, meningitis, encephalitis

## Abstract

**Background:**

Most enterovirus surveillance studies lack detailed clinical data, which limits their clinical usefulness. This study aimed to describe the clinical spectrum and outcome of severe enterovirus infections in children, and to determine whether there are associations between causative enterovirus genotypes and clinical phenotypes.

**Methods:**

Retrospective analysis of microbiological and clinical data from a tertiary children’s hospital in the South of England over a 17-month period (2012–2013).

**Results:**

In total, 30 patients were identified, comprising sepsis (n = 9), myocarditis (n = 8), meningitis (n = 8) and encephalitis (n = 5). Cases with sepsis or myocarditis were significantly younger than those with central nervous system disease (median age 21 and 15 days vs. 79 days; *P* = 0.0244 and *P* = 0.0310, respectively). There was considerable diversity in the causative genotypes in each of the clinical phenotypes, with some predominance of echoviruses in the meningitis group, and coxsackie B viruses in the myocarditis group. Thirteen cases required mechanical ventilation, 11 cases inotropic support, 3 cases dialysis and 3 cases extracorporal membrane oxygenation. The overall mortality was 10% (sepsis group, n = 1; myocarditis group, n = 2). Of the survivors, 5 (19%) had long-term sequelae (myocardial dysfunction, n = 2; neurological sequelae, n = 3). Patients with encephalitis had the longest hospital stay (median: 16 days), compared with 9, 6 and 3 days in patients with myocarditis, sepsis and meningitis, respectively (*P* = 0.005).

**Conclusions:**

Enterovirus infections, particularly enteroviral myocarditis and encephalitis, can cause significant morbidity and mortality. The results show that there are currently no strong associations between clinical phenotypes and particular causative enterovirus genotypes in the South of England.

Enterovirus infections in children are commonly asymptomatic or mild, but can present as severe disease, including sepsis, myocarditis, meningitis and encephalitis.^1^ These disease manifestations can be associated with significant morbidity and mortality, especially in neonates and young children.^2,3^

Data from previous publications suggest that certain enterovirus serotypes are associated with particular clinical phenotypes.^4–6^ However, most studies have been limited by the fact that they have primarily focused on one particular clinical phenotype [eg, myocarditis or central nervous system (CNS) disease] or on one particular enterovirus serotype.^4,7–12^ The vast majority of surveillance studies of enterovirus infections lack detailed clinical data, limiting their usefulness in the clinical context.^13^

Previous publications from both Europe and North America have shown that the predominant enterovirus serotypes change continuously over time.^3,14,15^ A recently published report describing epidemiological data of disease-causing enterovirus serotypes in England provided data on the predominant circulating serotypes, but only contained a very limited amount of clinical data, and therefore lacked the ability to identify potential associations between causative serotypes and clinical manifestations as well as disease severity.^16^

The aims of this study were to describe the clinical spectrum and outcome of severe enterovirus infections in children receiving care in a tertiary hospital setting in England, and to determine whether there are associations between causative enterovirus genotypes and clinical phenotypes.

## METHODS

A retrospective analysis was performed of microbiological and clinical data from children managed at the University Hospital Southampton NHS Foundation Trust (UHS). UHS provides services for a regional population of more than 600,000 children and adolescents in the South of England. The study captured data over a 17-month period (July 2012–November 2013). Children aged 0–18 years were included if (1) enterovirus was detected from any anatomical site, (2) their illness was severe enough to warrant hospital admission and (3) no definitive alternative diagnosis was established. Potential cases were identified from the diagnostic database of the Public Health England Southampton Regional Laboratory based at UHS.

For the detection of enteroviruses in clinical samples, a commercial real time-polymerase chain reaction (PCR) assay, Enterovirus R-gene (BioMérieux, Basingstoke, UK), was used. All enterovirus-positive samples were routinely genotyped at the Public Health England Virus Reference Unit in Colindale using previously described methods.^17^

Clinical data were extracted from the patients’ medical records into a standardized case report form, followed by transfer into an electronic database (Microsoft, Redmond, WA).

For analysis, cases were classified into clinical phenotypes using the following definitions^13^: (1) Hand-foot-mouth disease: mouth ulcers plus vesicular lesions on hands, feet, knees or buttocks, (2) Sepsis: systemic inflammatory response in the context of suspected or proven infection, according to published international pediatric consensus criteria,^18^ (3) Myocarditis: evidence of regional wall motion abnormalities or globally depressed left ventricular function identified by echocardiography or a creatinine kinase greater than 2 standard deviations above the upper limit of normal or unexplained arrhythmia,^19^ (iv) Meningitis: clinical features suggestive of meningitis and negative bacterial cultures and detection of enterovirus in cerebrospinal fluid (CSF), (v) Encephalitis: altered level of consciousness lasting more than 24 hours or focal neurological signs with abnormal electroencephalogram or cerebral imaging studies^20^ and (vi) “Unspecified” phenotype: clinical features not consistent with any of the clinical syndromes detailed above. In instances where cases matched more than one definition, they were categorized according to the predominant clinical phenotype.

In this article, the term “enterovirus CNS disease” is used as a collective term for enterovirus meningitis and enterovirus encephalitis. The term “circulatory compromise” is used to encompass children requiring fluid resuscitation and/or presenting with hypotension. CSF pleocytosis was defined as a CSF white blood cell (WBC) count >19/μL on microscopy during the first month of life, and >9/μL thereafter.^21,22^ Abnormal CSF protein level was defined as a protein concentration >500 mg/L. Normal values for biochemical parameters were defined as follows: C-reactive protein <5 mg/L, alanine transaminase (ALT) 3–35 IU/L and creatinine 20–70 μmol/L.

Statistical analyses were performed with Prism version 6.03 (GraphPad Software, La Jolla, CA). Kruskal–Wallis tests were used for the analysis of continuous data across multiple groups. If significant differences were detected, Mann–Whitney *U* tests were used for additional 2-group comparisons. To compare categorical data across multiple groups, *χ*^2^ tests were used.

The study was approved by the NHS Research Ethics Committee (approval no. 14/WA/1024) and the NHS Health Research Authority Confidentiality Advisory Group (approval no. 14/CAG/1014).

## RESULTS

**FIGURE 1.**
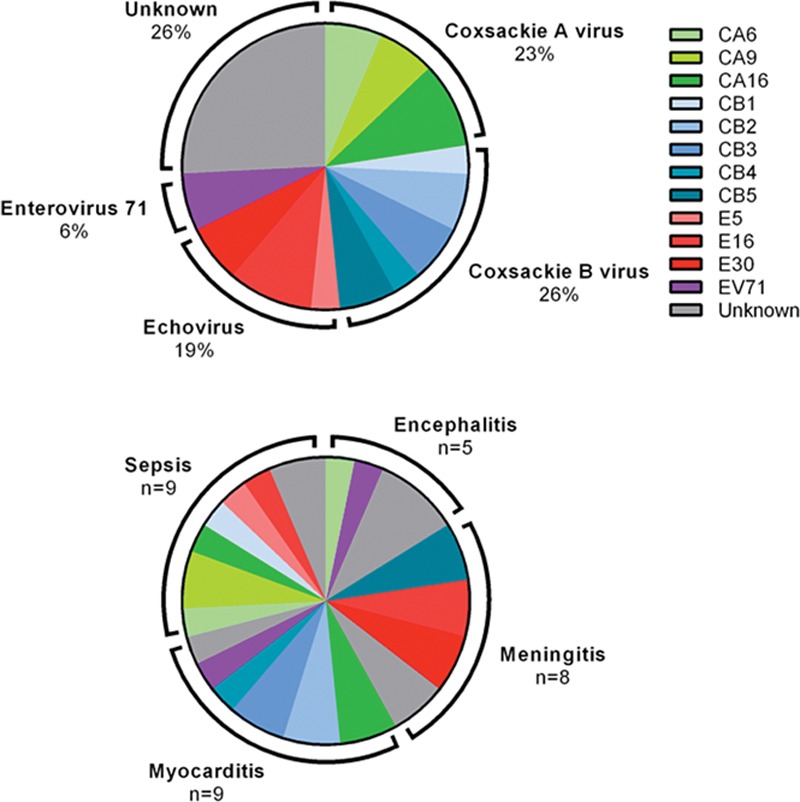
Causative enterovirus genotypes in the entire cohort (*upper panel*) and according to clinical phenotype (*lower panel*). In one patient with myocarditis, 2 enterovirus genotypes were identified. “Unknown” includes samples where typing was not performed or the virus was untypable. CA indicates coxsackie A virus; CB, coxsackie B virus; E, echovirus; EV, enterovirus.

A total of 30 patients hospitalized with severe enterovirus disease were identified and included in the final analyses. Among these cases, enterovirus was detected in blood (n = 10), CSF (n = 10), throat swabs (n = 15), rectal swabs (n = 17), skin swabs (n = 1) or unspecified tissue (n = 1; Table 1). In 19 (63%) patients, enterovirus was detected at 2 or more sites. The distribution of enterovirus genotypes in the entire study population is shown in Figure 1.

**Table 1.**
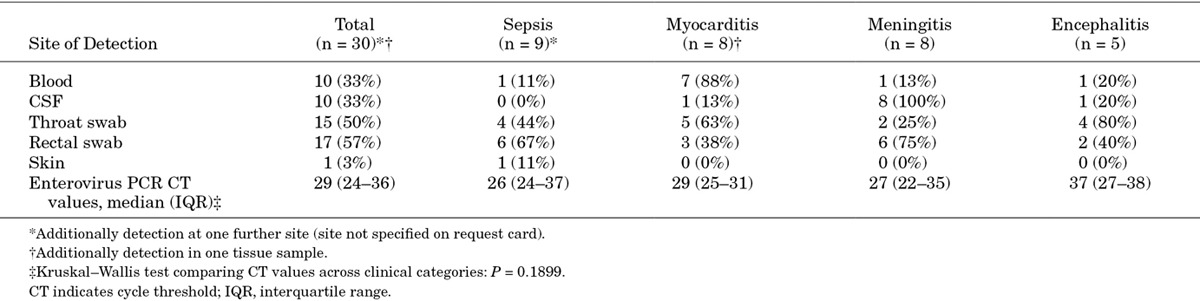
Association Between Clinical Phenotype and the Anatomical Sites from Which Enterovirus Was Detected

**FIGURE 2.**
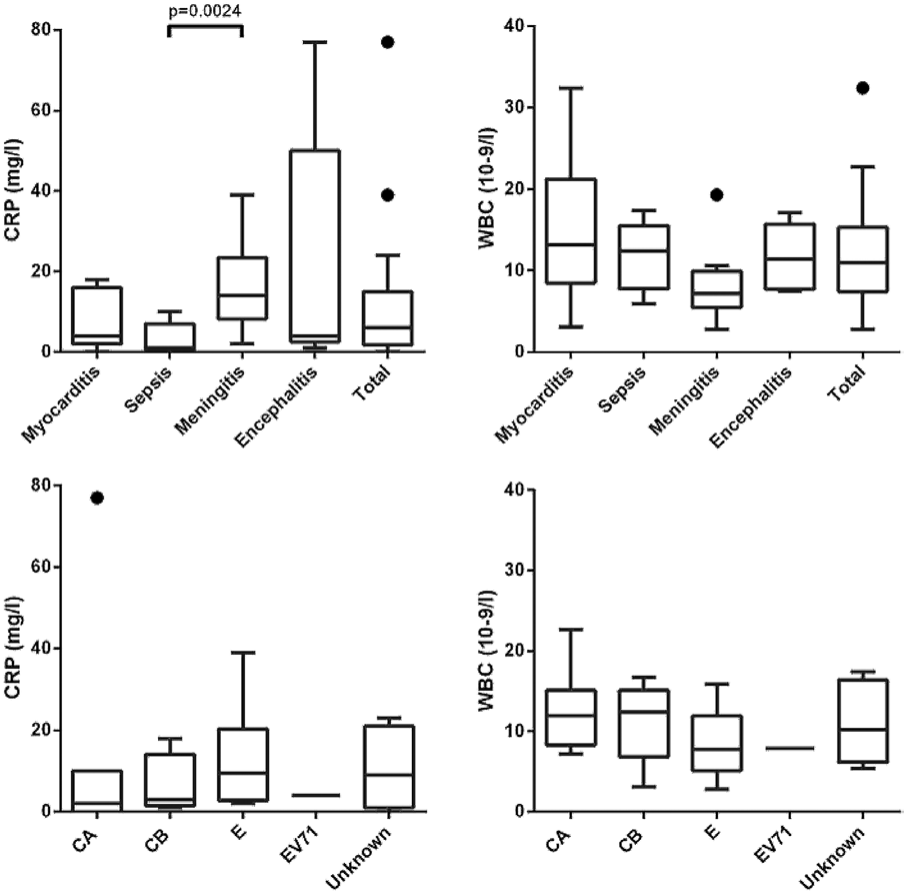
C-reactive protein and WBC at presentation according to enterovirus clinical phenotype (*upper panel*) and causative enterovirus genotype (*lower panel*). CA indicates coxsackie A virus; CB, coxsackie B virus; E, echovirus; EV, enterovirus.

The baseline clinical characteristics and laboratory results at presentation according to clinical phenotype are summarized in Table 2 and Figure 2. There were no statistically significant differences between the 4 phenotypic groups except for age and C-reactive protein concentration at presentation. Children presenting with sepsis or myocarditis were significantly younger than those presenting with CNS disease (median age 21 and 15 days vs. 79 days; *P* = 0.0244 and *P* = 0.0310, respectively). No children with hand-foot-mouth disease or an “unspecified” phenotype were admitted during the study period.

**Table 2.**
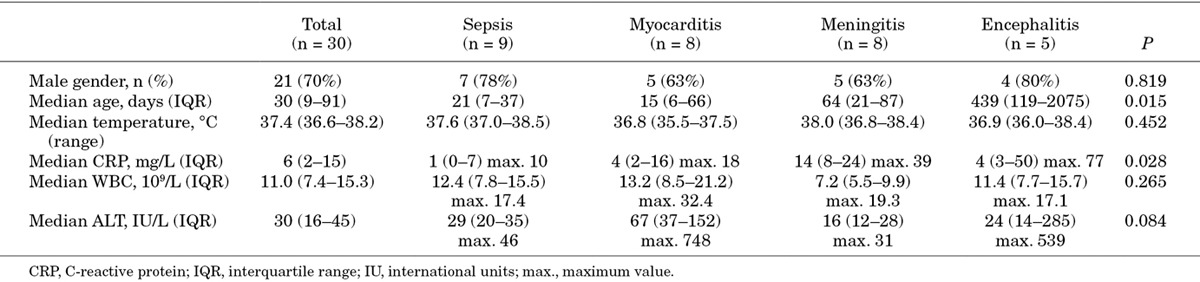
Baseline Characteristics at Presentation According to Clinical Phenotype

A lumbar puncture (LP) was performed in 23 patients; 15 of the CSF results were abnormal (pleocytosis, n = 12; elevated protein, n = 12; both, n = 9); 3 LPs were traumatic, as indicated by elevated CSF red blood cell counts.

A total of 13 patients required endotracheal intubation and ventilation. Twelve patients had circulatory compromise, of whom 11 required inotropic support; 3 additionally required extracorporeal membrane oxygenation (ECMO). Three patients requiring inotropic support had to be treated with dialysis as a result of acute renal failure.

### Sepsis

Nine patients fulfilled the definition for sepsis. Three patients had circulatory compromise, 1 of whom needed ventilation. The ALT concentration was mildly elevated (46 IU/L) in 1 patient. In 6 out of 8 cases who had undergone an LP CSF WBC count and/or protein concentration were elevated, but the enterovirus PCR performed on CSF was negative in all cases and the patients did not fulfill the criteria for encephalitis. The causative genotypes in those 6 cases comprised the following: coxsackie A16 (CA16), coxsackie B1 (CB1), echovirus 5 (E5), echovirus 16 (E16); 2 were not typed. One 9-day-old infant with enterovirus sepsis had an out-of-hospital arrest. He had focal seizures with secondary generalization on arrival in the emergency department, had circulatory compromise and required endotracheal intubation and ventilation. He died on the pediatric intensive care unit on day 6 of his admission. The postmortem examination did not identify a definite cause of death. A nontypable enterovirus was detected on a throat swab.

### Myocarditis

Of the 8 patients with myocarditis 1 presented with recurrent supraventricular tachycardia and pericardial effusion; the remaining 7 (88%) presented with circulatory compromise and required intubation and ventilation. The ALT concentration was elevated in 6 patients (75%), ranging from 42 to 748 IU/L. An LP was performed in 2 patients; both were traumatic. In 1 patient with myocarditis enterovirus 71 (EV71) was detected on a rectal swab and coxsackie B3 (CB3) was detected on a throat swab; the blood and CSF samples were also positive for enterovirus (not typed). This patient was severely unwell at presentation, and required ECMO support. Two patients with myocarditis, aged 6 and 7 days respectively at presentation, died despite ECMO support; the causative genotypes were CB3 and coxsackie B4 (CB4). At follow-up, 2 of the 6 survivors continued to have significantly reduced myocardial function; the remaining 4 made a full recovery.

### Meningitis

Eight patients fulfilled the criteria for enterovirus meningitis; as per the case definition, in all 8 cases enterovirus was detected in the CSF. One of these patients required intubation and ventilation, as well as inotropic support. None of these cases had abnormal focal neurological signs or seizures. The ALT concentration was normal in all patients. CSF results were abnormal in 7 patients and normal in 1 patient. None of these patients had evidence of neurological sequelae at discharge.

### Encephalitis

Five children fulfilled the study criteria for encephalitis, of whom 2 presented with focal seizures. CSF protein concentration and WBC results were normal in 3 and abnormal in 2 patients. In 1 case, the CSF PCR was positive for enterovirus. Computer tomography imaging of this patient showed bithalamic low attenuation signals consistent with encephalitis. The ALT concentration was abnormal in 1 of these patients (539 IU/L). One patient admitted after an out-of-hospital cardiac arrest was subsequently diagnosed with encephalitis based on electroencephalogram abnormalities. Long-term sequelae at follow-up in the encephalitis group comprised developmental delay (n = 1), reduced visual fields (n = 1) and persistent seizure disorder with focal features (n = 1).

### Duration of Hospital Stay and Survival

The median duration of hospital stay was significantly different between the 4 groups (Kruskal–Wallis *P* = 0.005). Patients with encephalitis had a median hospital stay of 16 days, while the duration of hospital stay in myocarditis, sepsis and meningitis patients was 9, 6 and 3 days, respectively. The overall mortality among the study population across all clinical phenotypes was 10% (3/30).

## DISCUSSION

This study demonstrates the spectrum of severe enterovirus disease in children hospitalized at a tertiary pediatric hospital serving a large regional population. The data show that enterovirus infections cause significant morbidity and mortality. Although this study a priori only included patients requiring admission to hospital, it is striking that the mortality in this cohort was substantial despite the availability of high-level PICU support, including ECMO. Also, it is notable that a considerable proportion of the survivors in the myocarditis group had persistently reduced myocardial function, which is concordant with the findings of a recent study from The Netherlands.^23^

Our data show that children presenting with enterovirus sepsis or myocarditis are significantly younger on average than those presenting with enterovirus CNS disease. Considering that almost all cases with sepsis and myocarditis (15 out of 17 patients) in this study presented within the first 3 months of life, we believe that all young infants presenting with fever and circulatory compromise or other symptoms consistent with sepsis should be tested for enterovirus infection. Interestingly, we found that only in a relatively small proportion of cases (11%) in the sepsis group enterovirus could be detected in blood, while rectal swabs had a much higher yield (67%) in this group. This is consistent with observations from a previous study in young infants with enterovirus sepsis, which found that real-time PCR cycle threshold values are generally considerably lower in fecal samples than in blood samples.^24^ Timely testing for enteroviruses can help to inform management decisions, including the consideration to discontinue broad-spectrum antibiotics in these patients. Unfortunately, current treatment options for enterovirus infections are limited as there are no licensed antiviral treatment options.^2,25,26^ Also, the use of intravenous immunoglobulin for severe enterovirus infections, including myocarditis, remains controversial as the currently available data are insufficiently robust.^2^

The results also highlight that there is considerable diversity in the causative genotypes in each of the clinical phenotypes. However, there was a relative predominance of echoviruses in patients with meningitis, and some predominance of coxsackie B viruses in the myocarditis group. The latter association has been described in previous publications.^4,23,27,28^

Earlier studies have shown that the predominant enterovirus serotypes within populations change continuously over time.^3,14,15^ In our study, we found that coxsackie A viruses, coxsackie B viruses and echoviruses accounted for similar proportions of the severe enterovirus infections. This contrasts with data from an epidemiological study conducted in England and Wales that reported data from 1975 to 1994, in which 61% of the culture-confirmed enterovirus isolates were found to be echoviruses, 10% coxsackie A viruses and 29% coxsackie B viruses.^29^ A recently published study providing laboratory data from the national reference laboratory in England and Wales from the period 2000 to 2011 reported similar proportions, with echoviruses accounting for 55%, coxsackie A viruses for 5%, and coxsackie B viruses for 23% of the enterovirus strains for which typing result were available.^16^ Comparing our data to those national data it appears that cases with coxsackie A virus infection were relatively overrepresented in our cohort, although the significance of this remains uncertain. However, our data show that this was not due to a single coxsackie A virus serotype with particular virulence.

In one patient with encephalitis two potentially causative organisms, an enterovirus and an adenovirus, were detected in respiratory secretions. The cause of the CNS disease in this case therefore remains uncertain. Published data, including large-scale epidemiological studies, suggest that enterovirus-related CNS disease is far more common than adenovirus-related CNS disease.^30^ Also, in contrast to enteroviruses, adenoviruses have a propensity to persist in lymphoepithelial tissues and detection of this organism may therefore merely have reflected past infection in this patient.

Two additional findings related to CNS disease are intriguing and warrant further investigation. First, our data show that in some patients with clinical features of meningitis in whom enterovirus is present in the CSF, CSF WBC and protein concentrations are within normal limits. This highlights that an unremarkable CSF analysis does not rule out enterovirus meningitis, an observation that is consistent with a recent small study from Scotland, which focused exclusively on children with enterovirus sepsis and CNS disease.^9^ Second, our results show that in a considerable proportion of cases with enterovirus infections who present with sepsis-like symptoms (and without clinically apparent meningitis), the CSF analysis is abnormal.

The main limitation of our study lies in the comparatively small sample size, resulting from the fact that the study was conducted at a single healthcare institution. However, this is the first description of predominant causative enteroviruses causing severe neonatal and pediatric disease in England in more than a decade. It remains uncertain whether our data can be extrapolated to the rest of the United Kingdom. Consequently, a multicenter study investigating the correlation between clinical phenotypes and causative enterovirus genotypes would be desirable. Our study was deliberately designed to describe severe enterovirus disease in children admitted to a tertiary children’s hospital, and therefore does not provide data on “milder” forms of enterovirus disease that are primarily encountered in the community setting.

## CONCLUSION

Enterovirus infections, particularly enterovirus myocarditis and encephalitis, can result in significant morbidity and mortality. Even with high-level PICU support available, a substantial proportion of patients with severe enterovirus disease have a fatal outcome. In addition, our data show that there is considerable diversity in the enterovirus genotypes causing severe disease in the South of England, with coxsackie A virus-related disease being relatively overrepresented when compared with national epidemiological data. Furthermore, the results highlight that there are currently no strong associations between clinical phenotypes and particular causative enterovirus genotypes in the South of England.
